# Pathology Slide Review in Vulvar Cancer Does Not Change Patient Management

**DOI:** 10.1155/2014/385386

**Published:** 2014-03-25

**Authors:** Maaike Beugeling, Patricia C. Ewing-Graham, Zineb Mzallassi, Helena C. van Doorn

**Affiliations:** ^1^Department of Gynecological Oncology, Erasmus Medical Center, Rotterdam, Cancer Institute, P.O. Box 5201, 3008 AE Rotterdam, The Netherlands; ^2^Department of Radiation Therapy, Erasmus Medical Center, Cancer Institute, P.O. Box 5201, 3008 AE Rotterdam, The Netherlands; ^3^Department of Pathology, Erasmus Medical Center, P.O. Box 2040, 3000 CA Rotterdam, The Netherlands

## Abstract

*Hypothesis*. Pathology slide review in vulvar cancer is only necessary in a restricted number of cases. *Methods*. A retrospective chart review of all cases of vulvar cancer treated in a tertiary centre between January 1, 2000, and April 1, 2006. Histopathology reports from the referring and tertiary centre were compared. *Results*. 121 pathology reports from 112 patients were reviewed. Of the original reports, 56% were deemed adequate, commenting on tumor type and depth of infiltration; of the reviews, 83% were adequate. *Conclusion*. There were no discrepancies that influenced patient management. We suggest that vulvar cancer biopsies need to be reviewed only when the tumor is less than 10 mm in linear extension, when the infiltration is 1 mm or less, when there is no residual tumor on inspection, and in any nonsquamous cancer.

## 1. Introduction

Vulvar cancer accounts for 2–5% of gynecological cancers, and 90% of cases are squamous carcinoma. Tumor type and infiltration depth are crucial in determining treatment. Treatment of the groin nodes is recommended when infiltration depth is 1 mm or more or when tumor size is 2 cm or more (T1B tumors). In our institute, treatment of groin nodes consists of either a sentinel node procedure or an inguinal-femoral lymphadenectomy, depending on the size of the tumor and the number of tumor foci. Different treatment schedules are applicable for superficially invasive tumors (if smaller than 2 cm diameter) and for nonsquamous carcinomas. It is therefore clearly of importance that the biopsy report should, where possible, include tumor type and infiltration depth. These features, in combination with a clinical assessment of tumor diameter, should enable an appropriate decision about treatment to be made. In the Netherlands, patients with vulvar malignancies are referred to tertiary centers for treatment. In our tertiary institute the slides are reviewed by pathologists experienced in gynecopathology as part of the preoperative workup. The extra work and resources involved in reviewing all tumors are discussed and debated in several papers [1–5], although literature on this topic is relatively scarce. It is clear that review is costly and time consuming. Both laboratories have to organize the exchange of tissue blocks and slides, which is time consuming for administrative assistants. Reviewing slides costs time for the pathologist and the review may potentially delay treatment, increasing stress for patients and relatives. The objective of this paper is to determine the impact of pathology review on patient management in patients with vulvar cancer. In addition, we investigate the adequacy of the pathology reports, with regard to tumor type, infiltration depth, and, for excision biopsies, resection margins.

## 2. Materials and Methods

We conducted a retrospective chart review of all patients referred and treated for cancer of the vulva at Erasmus MC, Rotterdam, in the period from January 1, 2001, to April 1, 2006. Such patients areexamined by the gynecologist-oncologist at the outpatient department where the location of the tumor, its size, and the number of lesions are recorded. Review of the histology is requested in all cases and the slides are revised by a pathologist experienced in gynecopathology prior to surgery.

Information was drawn from the original and review pathology reports and from hospital charts. The data collected included patient characteristics, type of specimen (incisional versus excisional biopsy), biopsy site, histological type of the tumor, tumor grade, infiltration depth, and, for excisional biopsies, resection margins. Where patients had had several biopsies, each biopsy was classed as a separate review. Any extra histological biopsy that was required in the tertiary centre for proper treatment planning was recorded. Discrepancies between the original and review diagnoses were sought. A discrepancy was defined either as a difference in histological type or in measurement of infiltration depth, where one report stated that infiltration depth was less than 1 mm and the other stated that there was invasion to a depth of more than 1 mm (this being the critical cutoff for treatment of the groin nodes).

A histology report was considered “adequate” when it described tumor type, depth of infiltration, and—in excisional biopsies—resection margins. A report was regarded as “incomplete” if these parameters were not included. Sometimes the pathologist was unable for technical reasons to accurately measure depth of infiltration. If this problem was clearly described in the report we regarded this as being a comment on depth of infiltration. Statistics were done with SPSS 15.0. Pearson Chi-square test was used for the analysis.

## 3. Results

Patients were referred to our clinic from 19 hospitals, each referring between one and 24 patients. A total of 121 tumors in 112 patients were included. Mean age at biopsy was 71.9 years. The number of biopsies ranged from one to six with a mean of 1.4. In 93 patients, the tumor was unifocal and in 11 patients multifocal. In eight cases, this was not specified. Most biopsies (67.8%) were incisional biopsies.  Table 1 shows data on histological findings.

In one case, a diagnosis of carcinoma of the Bartholin gland was made in the original report while the review diagnosis was squamous carcinoma with infiltration depth of more than 1 mm. A third opinion was sought at another institute which confirmed our review report. In one further case, a third opinion was sought from an expert pathologist in another institute to confirm a rare mix of verrucous carcinoma and conventional squamous carcinoma. In three patients, it was necessary to rebiopsy, since the original biopsies were inconclusive regarding tumor type.

Our findings with regard to infiltration depth are shown in Table 2.

Where the depth of infiltration was missing from the original report, this was usually found to be >1 mm on review. There were 17 reviews where it was not possible to measure infiltration depth; in 16 of these cases the definitive vulvectomy specimen showed a depth of infiltration >5 mm. The remaining patient received palliative treatment and therefore there was no definitive specimen.

A report stating histological type and depth of infiltration was considered “adequate” for the purposes of this study. Using this criterion, 56% of the original reports were adequate. Of the review reports, 83% were adequate. Tumor grade was not consistently given in reports. There was no grade stated in 41 reports from the referring institutes and in 20 of the review reports.

## 4. Discussion

In our study on slide review in vulvar cancer we found only one instance where the management of the patient changed as a result of the pathology review. This was a case where a patient was referred with a tumor of a Bartholin cyst which on review was classified as a squamous carcinoma of the vulva. Carcinoma of Bartholin's gland is, however, not the same entity as vulvar carcinoma and treatment protocols differ. There were therefore no cases referred to our institute with squamous carcinoma of the vulva where management altered as a result of slide review.

In a large number of cases, the report was incomplete; in 44% of the original reports, a comment on depth of infiltration was missing. In the review reports, infiltration depth was encountered more frequently, but still missing in some cases.

Squamous carcinoma of the skin is very well-known to pathologists and vulvar carcinoma resembles other squamous carcinomas of the skin histologically; however, treatment differs markedly, and histological typing alone is insufficient for treatment planning in the case of vulvar tumors. Histopathologists need therefore to include the infiltration depth in their reports on squamous carcinoma from this site.

Our group of 121 biopsies is relatively large compared to other studies.  Table 3 shows an overview of the literature on slide review in vulvar cancer. The available literature on this topic shows a rate between 0% and 15.8% for major discrepancy. It is not possible from our study, or from the wider literature, to calculate how many histology reviews would be necessary to find one major discrepancy, nor the cost of this review process. Ideally these issues should be clarified by a cost-benefit study before protocols relating to the process of referral and treatment in tertiary centers are altered. We doubt however whether slide review is necessary for vulvar cancer, and this view is shared by other authors [3, 5]. Based on the findings of this study, we have altered the protocol for our clinic. We now request slide review only in specific circumstances: when the tumor is less than 10 mm in linear extension, when the infiltration depth is 1 mm or less, when there is no residual tumor on inspection, and for any tumor described as nonsquamous cancer. For excision biopsies, resection margins should be clearly stated. In addition, pathologists in the referring clinics are requested to include infiltration depth, the presence of capillary-lymphatic space invasion, and width of resection margins in their report.

## Figures and Tables

**Table 1:** Histological findings.

	Original report, *n* = 121	Review report, *n* = 121
Histological type		
Squamous cell	113	114
Adenocarcinoma	3	3
Not specified	1	1
Other types of epithelial tumor*	4	3
Tumor grade		
Unknown	41	20
Well differentiated	37	40
Moderately differentiated	38	53
Poorly differentiated	5	8
Depth of infiltration		
Less than 1 mm	5	4
More than 1 mm	52	79
Not possible	11	17
Missing	53	21
Margins (only for excisions, *n* = 39)		
Less than 1 cm	26	24
More than 1 cm	5	8
Missing	8	7
Completeness of report		
Adequate**	68 (56%)	100 (83%)
Incomplete	53 (44%)	21 (17%)

*Including a mixed tumor with squamous carcinoma and adenocarcinoma, a granular cell tumor, and an adenoid cystic carcinoma.

**Adequate reports include tumor type and infiltration depth.

**Table 2:** Infiltration depth in original and review reports.

Original/review	<1 mm	>1 mm	Not assignable	Missing
<1 mm	2	1	0	2
>1 mm	0	45	2	5
Not assignable	1	4	6	0
Missing	1	29	9	14

**Table 3:** Overview of literature on pathology slide review in vulvar cancer.

	Gynecologic oncologic reviews	Vulva reviews		
		Number	Minor discrepancies (%)	Major discrepancies (%)
Santoso et al. 1998 [1]	720	113	n.s.	1 (0.88)
Chan et al. 1999 [2]	569	13	2 (15.4)	2 (15.4)
Selman et al. 1999 [3]	295	19	0	3 (15.8)
Chafe et al. 2000 [4]	599	28	9 (33)	2 (7)
Khalifa et al. 2003 [5]	351	28	3 (10.7)	0
Beugeling	121	121	0	2 (1.6)

n.s.: not specified.
